# Accuracy of SD Malaria Ag P.f/Pan® as a rapid diagnostic test in French Amazonia

**DOI:** 10.1186/s12936-021-03902-z

**Published:** 2021-09-17

**Authors:** Jean Marc Pujo, Stéphanie Houcke, Sarah Lemmonier, Patrick Portecop, Alexis Frémery, Denis Blanchet, Felix Djossou, Hatem Kallel, Magalie Demar

**Affiliations:** 1 Emergency Department, Cayenne General Hospital, Cayenne, French Guiana; 2 Intensive Care Unit, Cayenne General Hospital, Cayenne, French Guiana; 3 Emergency Department, Guadeloupe University Hospital, Les Abymes, Guadeloupe; 4 Academic Laboratory of Parasitology-Mycology, Cayenne General Hospital, Cayenne, French Guiana; 5 Tropical Biome and Immunophysiopathology (TBIP), Université de Guyane, Cayenne, 97300 French Guiana; 6 Université de Lille, CNRS, Inserm, Institut Pasteur de Lille, U1019-UMR9017-CIIL Centre d’Infection Et d’Immunité de Lille, 59000 Lille, France; 7 Infectious and Tropical Diseases Unit, Cayenne General Hospital, Cayenne, French Guiana

**Keywords:** Malaria, Rapid diagnosis tests, Accuracy, PfHRP2 gene deletion

## Abstract

**Background:**

French Guiana (FG) is a French overseas territory where malaria is endemic. The current incidence rate is 0.74‰ inhabitants, and *Plasmodium vivax* is widely predominating even though *Plasmodium falciparum* is still present due to imported cases mainly from Africa. In FG, rapid diagnostic test (SD Malaria Ag P.f/Pan®) is based on the detection of pan-pLDH, PfHRP2, and PfHRP3 antigens, while in South America, the share of deletion of PfHRP2 gene is significantly increasing. Accordingly, the study questions the reliability of RDTs in the Amazonian context.

**Methods:**

The study is retrospective. It is conducted over 4 years and analysed 12,880 rapid diagnostic tests (RDTs) compared to concomitant Blood Film Tests (BFTs) sampled for malaria diagnosis.

**Results:**

The global assessment of the accuracy of SD Malaria Ag P.f/Pan® in the diagnostic of malaria shows both Positive and Negative Predictive Values (PPV and NPV) higher than 95%, except for PPV in the diagnosis of malaria to *P. falciparum* (88%). Overall, the concordance rate between RDT and BFT (positive/positive; negative/negative) was 99.5%. The PPV of the RDT in the follow-up of patients diagnosed with *P. falciparum* was the lowest during the first 28 days. The PPV of the RDT in the follow-up of patients diagnosed with *P. vivax* was the lowest during the first 21 days. The global sensitivity of SD Malaria Ag P.f/Pan® test was, on average, 96% (88.2–100) for *P. falciparum* and 93% (90.6–94.2) for *P. vivax*. The global specificity was 99.8% (99.5–100) for all included species.

**Conclusion:**

SD Malaria Ag P.f/Pan® is a reliable rapid test used for the first-line diagnosis in remote healthcare centres. The test results should be interpreted in the light of patient’s recent medical history and the date of arrival to FG.

## Background

WHO classified malaria as a public health concern. In 2017, malaria was the most prevalent parasitic disease, with 1.4 billion people at risk worldwide [1]. The year after, the number of malaria cases was estimated at 228 million, with 405,000 recorded deaths worldwide, mostly in the African continent. In the Americas, the malaria incidence has been increasing since 2016, mainly due to the epidemic situation in Venezuela [2, 3] with 75% of cases caused by *Plasmodium vivax* [2]. In French Guiana (FG) malaria is endemic [4]. Since the 1950s, efforts to fight malaria coupled with the control strategies implemented in the Guiana Shield [5] have significantly reduced the incidence of the disease [6]. Indeed, the malaria incidence recorded in 2019 in FG was the lowest during the ten previous years (0.74‰ inhabitants) [7].

Previously in FG, *Plasmodium falciparum* was accountable for the majority of cases. However, within the last 20 years, the distribution of *Plasmodium* species changed with a vast rate of diagnosed *P. vivax* [6, 8]. Despite a very encouraging figure with a very low malaria transmission rate in the coastal and urban areas, the risk of malaria remains endemic in FG. The country's inland sites continue to experience high levels, mainly in socially marginalized and isolated populations.

Among laboratory methods used for malaria parasite detection and exposure, only rapid diagnostic tests (RDT) and blood films tests (BFT) are currently available for daily practice. Molecular detection of low-density *P. falciparum* infections is a crucial point for surveillance studies to steer malaria control strategies in countries where malaria is near to almost elimination [9]. RDT is based on the detection of pan-pLDH, PfHRP2 and PfHRP3 antigens. However, there is an increasing rate of deletion of the PfHRP2 gene worldwide, threatening the ability to diagnose patients infected with *P. falciparum*, and causing false negative RDT results [10]. In South America, the distribution of PfHRP2 gene deletion varies with free countries and areas where the deletion rate is over 30% [11]. In FG, the RDT used is SD Malaria Ag P.f/Pan® and *P. falciparum* can present PfHRP3 deletion (4.5% of cases) but no PfHRP2 deletion [12].

This study aims to assess the relevance of SD Malaria Ag P.f/Pan® in the diagnosis of malaria in French Guiana.

## Methods

Our study is a retrospective analysis conducted over 4 years (January 2016 to December 2019) in the microbiology laboratory of the Cayenne General Hospital. It includes all RDT and BFT sampled for malaria diagnosis. Cayenne General Hospital is a 742-beds health facility that provides first-line medical care for an urban population of 150,000 inhabitants. It manages 18 delocalized prevention and healthcare centers providing care for almost 50,000 inhabitants. Thereby, it is also a referral centre for a larger population from all over French Guiana and other border countries [13].

### Data collection

Data were collected from the computerized database of the microbiology laboratory of the Cayenne General Hospital. They include the date of the tests, and the results of the RDT and the concomitant BFT. In a first step, files with a RDT and a concomitant BFT were included and files with a BFT without a concomitant RDT were excluded. Also files where concomitant RDT/BFT were sampled during the six months following the malaria diagnosis were separately analysed.

### Microbiological technique

RDT was based on the SD Malaria Ag P.f/Pan® (Standard Diagnostics Inc.) which detects the presence of pan-pLDH and PfHRP2 antigens [12]. BFT is based on microscopic examination of blood and represents the gold standard for the malaria diagnosis [14]. Two sorts of blood film are traditionally used. Thick films allow the screening of a larger blood volume and are about eleven times more sensitive than thin films. It enables the diagnosis of infection with a low level of parasites. In contrast, thin films allow better identification of the responsible parasite. Both smears are recommended when attempting to make a definitive diagnosis of malaria [15].

Thick and thin blood films are prepared within one hour of blood collection. Thick blood films are stained with Giemsa diluted at 10%, while thin blood films are stained using a rapid method (RALH 555, RAL Diagnostics). Two hundred fields of the thin blood film are examined before classifying the thin smear-negative, and 1000 counted white blood cells (WBCs) from the thick smear are observed before classifying the sample as negative [12]. The parasite density estimation is based on an assumed 6000 WBC/ml of blood [12].

### Statistical analysis

Results are reported as mean and standard deviation, or numbers and percentages. We calculated the sensitivity (Ss), specificity (Sp), positive and negative predictive values (PPV and NPV), Youden test, and the Q coefficient of Yule to assess the diagnostic accuracy of RDT in the diagnosis of confirmed malaria by BFT. All statistical analyses were carried out using Excel (2010 Microsoft corporation, Redmond, USA) and IBM SPSS Statistics for Windows, version 24 (IBM Corp., Armonk, NY, USA).

### Ethical consideration

The study is retrospective that did not require individual consent according to the French law regarding research conforming to MR-003 (JORF no. 0160 du 13 juillet 2018. texte no. 109). The database has been registered at the Commission National de l'Informatique et des Libertés (registration no 2219819), in compliance with French law on electronic data sources.

## Results

**Fig. 1:**
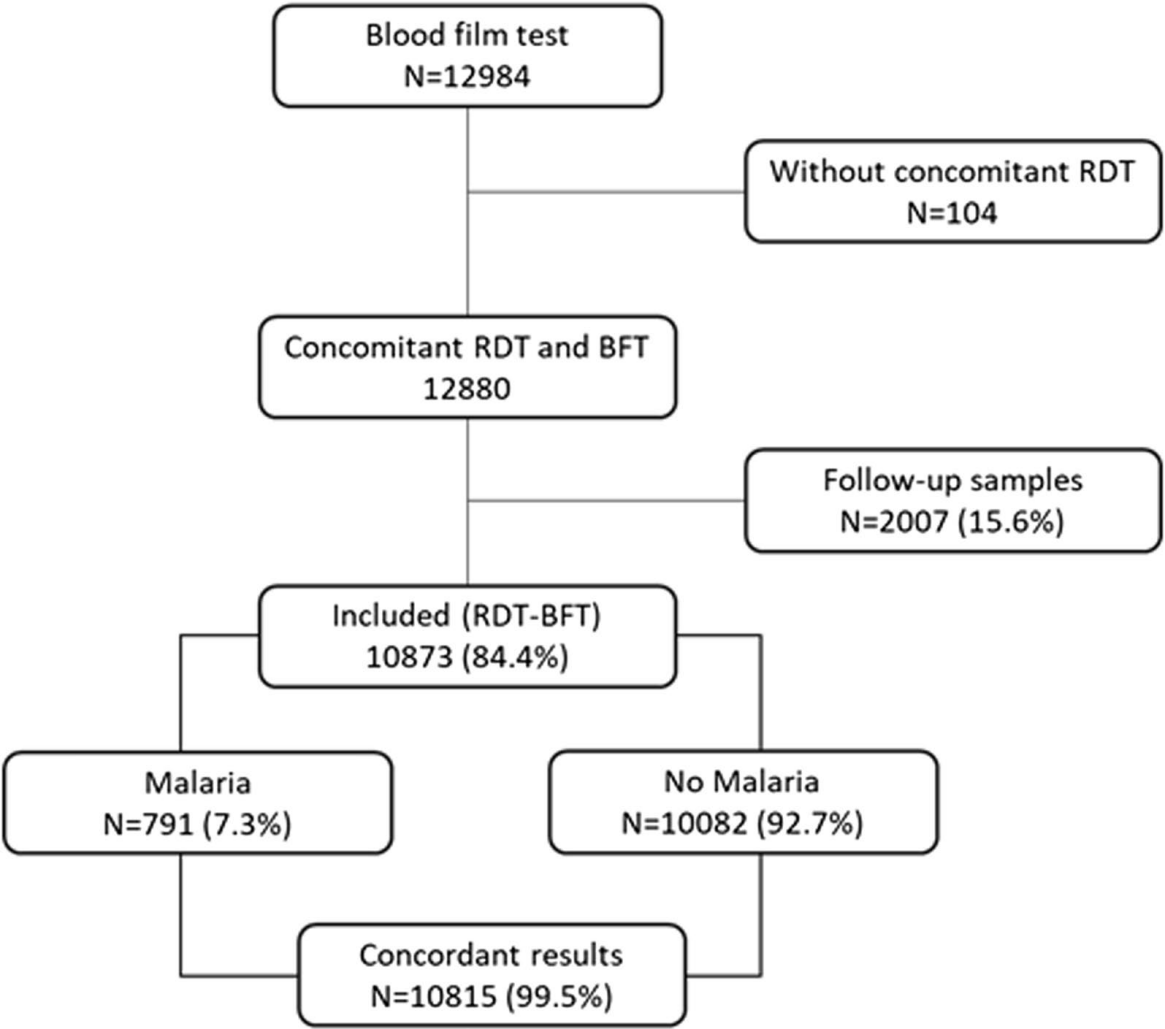
The flow chart of the study (N = Number of cases)

During the study period, 12,984 samples of blood films for the diagnostic of malaria were recorded. A concomitant BFT and RDT were sampled in 12,880 cases. Amongst them, 10,873 files (84.4%) fulfilled our inclusion criteria (Fig. 1).

The average number of RDTs and BFTs per year was 2718 ± 394 tests. The average number of RDTs and BFTs per month was 1073 ± 105 tests. RDT was performed in 10,873 cases and was positive in 773 cases (7.1%) with identification of *P. falciparum* in 125 cases (16.1% of positive tests) and *P. vivax* in 648 cases (83.9% of positive tests). BFT was performed in 10,873 cases and was positive in 791 cases (7.3%) with identification of *P. falciparum* in 105 cases (13.3% of positive tests), *P. vivax* in 673 cases (85.1% of positive tests), both of them in 10 cases (1.3% of positive tests), and *Plasmodium ovale* in 3 cases (0.4% of positive tests).

**Fig. 2:**
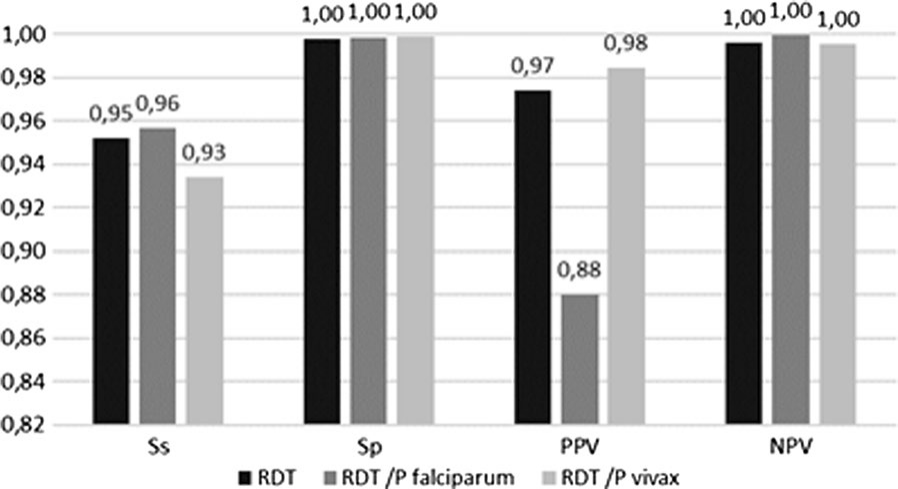
The diagnosis accuracy of RDT in diagnosing malaria according to the *Plasmodium* species identification. (RDT: Rapid diagnosis tests, Ss: Sensitivity, Sp: Specificity, PPV: Positive Predictive Value, NPV: Negative Predictive Value)

The global assessment of the accuracy of RDT in the diagnosis of malaria shows an overall PPV and NPV higher than 95%, except for PPV in the diagnosis of malaria to *P. falciparum* (88%). Figure 2 shows the accuracy of RDT in the diagnosis of malaria independently of the *Plasmodium* specie*s* identification.

**Fig. 3:**
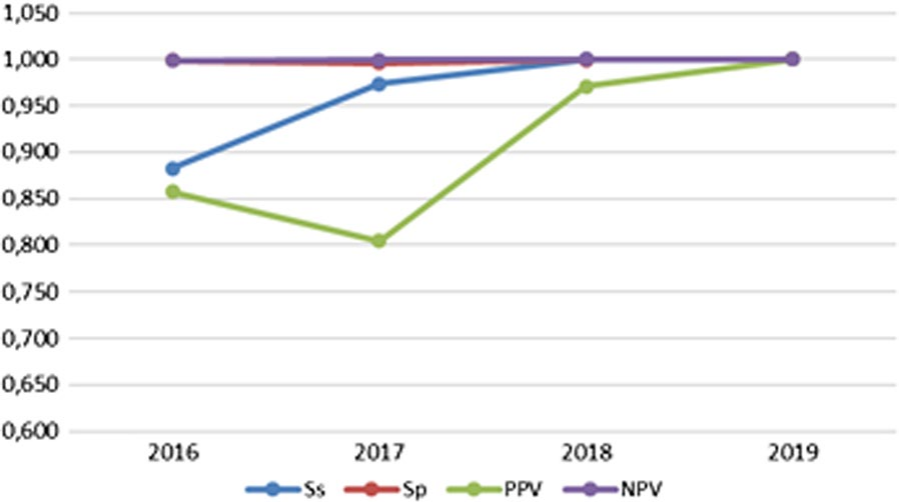
The diagnosis accuracy of RDT in the diagnosis of *Plasmodium falciparum* according to the year of the study. (RDT: Rapid diagnosis tests, Ss: Sensitivity, Sp: Specificity, PPV: Positive Predictive Value, NPV: Negative Predictive Value)

The yearly assessment of the accuracy of RDT in the diagnosis of malaria to *P. falciparum* and *P. vivax* is reported in Table 1. The yearly assessment of the accuracy of RDT in the diagnosis of malaria to *P. falciparum* shows a drop-down in the PPV in 2017, but an increase after that (Fig. 3).

**Table 1:** The yearly accuracy of RDT in the diagnosis of malaria

Year	Comparaison variable	Nb	TP	FP	TN	FN	Ss	Sp	PPV	NPV	Q	Youden
2016	RDT	3276	104	6	3164	2	0.981	0.998	0.945	0.999	1.000	0.979
	RDT /*P. falciparum*		30	5	3237	4	0.882	0.998	0.857	0.999	1.000	0.881
	RDT /*P. vivax*		71	4	3196	5	0.934	0.999	0.947	0.998	1.000	0.933
2017	RDT	2462	297	10	2144	11	0.964	0.995	0.967	0.995	1.000	0.960
	RDT /*P. falciparum*		37	9	2415	1	0.974	0.996	0.804	1.000	1.000	0.970
	RDT /*P. vivax*		258	3	2185	16	0.942	0.999	0.989	0.993	1.000	0.940
2018	RDT	2714	265	3	2430	16	0.943	0.999	0.989	0.993	1.000	0.942
	RDT /*P. falciparum*		33	1	2680	0	1.000	1.000	0.971	1.000	1.000	1.000
	RDT /*P. vivax*		232	2	2464	16	0.935	0.999	0.991	0.994	1.000	0.935
2019	RDT	2421	87	1	2324	9	0.906	1.000	0.989	0.996	1.000	0.906
	RDT /*P. falciparum*		10	0	2411	0	1.000	1.000	1.000	1.000	1.000	1.000
	RDT /*P. vivax*		77	1	2335	8	0.906	1.000	0.987	0.997	1.000	0.905

RDT: Rapid diagnosis tests, Nb: Number of cases, TP: True positive, FP: False positive, TN: True negative, FN: False negative, Ss: Sensitivity, Sp: Specificity, PPV: Positive Predictive Value, NPV: Negative Predictive Value

**Fig. 4:**
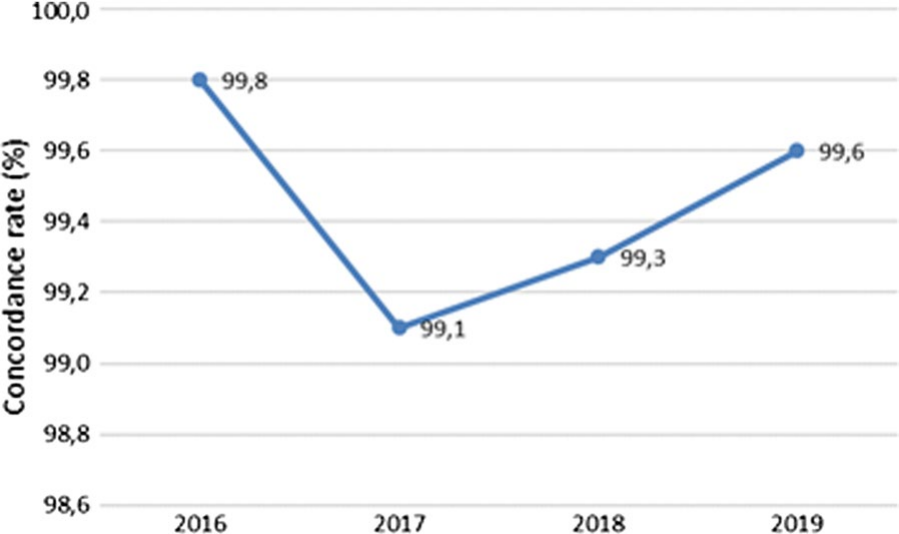
The concordance rate of RDT compared to BFT in the diagnosis of malaria independently of the plasmodium species identification according to the year of the study

Overall, the concordance rate between RDT and BFT (positive/positive; negative/negative) was 99.5%. It was the highest in 2016 (99.8%) and the lowest in 2017 (99.1%) (Fig. 4). The concordance rate between RDT and BFT in the diagnosis of *P. falciparum* was 99.8%. It was at 99.6% in 2017 (the lowest) and 100% in 2018–2019. The concordance rate between RDT and BFT in the diagnosis of *P. vivax* was 99.3%. It was at 99.1% in 2017 (the lowest) and 99.5% in 2016 and 2019.

**Fig. 5:**
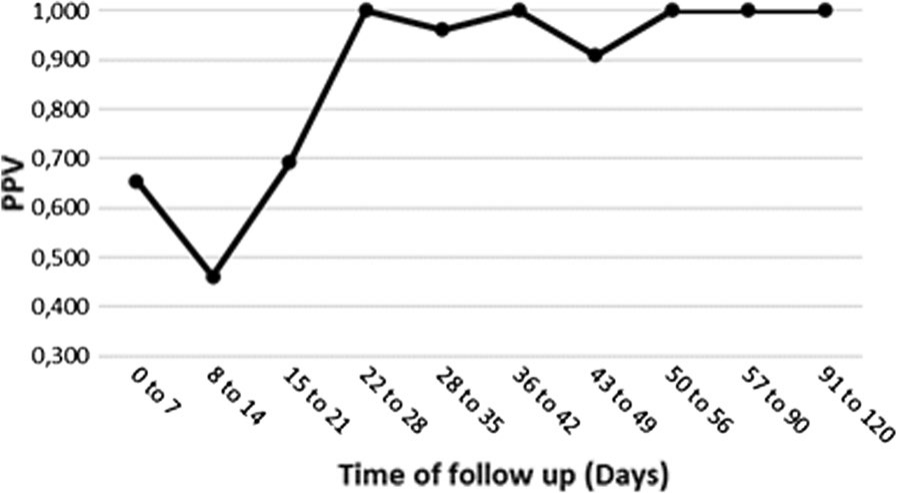
Diagnosis accuracy of RDT in the follow-up period of malaria independently of the *Plasmodium* species identification

During the study period, RDTs and BFTs were performed as part of the follow-up (in patients previously diagnosed with malaria) in 2007 cases (15.6%). The PPV value of RDT in this context was the lowest during the 42 days of follow-up (Fig. 5). The PPV of the RDT in the follow-up of patients diagnosed with *P. falciparum* was the lowest during the 28 first days. The PPV of the RDT in the follow-up of patients diagnosed with *P. vivax* was the lowest during the 21 first days (Table 2).

**Table 2:** The accuracy of RDT during 4 months of follow-up after the diagnosis of malaria (all species included)

Time (days)	Nb	TP	FP	TN	FN	Ss	Sp	PPV	NPV	Q	Youden
0 to 7	992	116	61	803	12	0.906	0.929	0.655	0.985	0.984	0.836
8 to 14	221	6	7	208	0	1.000	0.967	0.462	1.000	1.000	0.967
15 to 21	123	9	4	109	1	0.900	0.965	0.692	0.991	0.992	0.865
22 to 28	91	20	0	70	1	0.952	1.000	1.000	0.986	1.000	0.952
28 to 35	76	25	1	48	2	0.926	0.980	0.962	0.960	0.997	0.906
36 to 42	64	19	0	44	1	0.950	1.000	1.000	0.978	1.000	0.950
43 to 49	46	10	1	35	0	1.000	0.972	0.909	1.000	1.000	0.972
50 to 56	33	7	0	26	0	1.000	1.000	1.000	1.000	1.000	1.000
57 to 90	151	29	0	119	3	0.906	1.000	1.000	0.975	1.000	0.906
91 to 120	86	14	0	72	0	1.000	1.000	1.000	1.000	1.000	1.000

RDT: Rapid diagnosis tests, Nb: Number of cases, TP: True positive, FP: False positive, TN: True negative, FN: False negative, Ss: Sensitivity, Sp: Specificity, PPV: Positive Predictive Value, NPV: Negative Predictive Value

## Discussion

The objective of the study was to assess the accuracy of SD Malaria Ag P.f/Pan® test, which is the RDT currently used in remote healthcare centers in FG, in the rapid diagnosis of malaria. The SD Malaria Ag P.f/Pan® test was sufficiently accurate in diagnosing malaria in suspected patients, in routine monitoring, and in detecting passive cases in malaria low transmission areas [16, 17]. Please move to references Rapid diagnosis of malaria is essential to introduce early curative treatment and prevent a severe outcome. High-quality RDTs may be used, as a first measure, thanks to their efficiency, large availability, and low cost [18]. In FG remote areas, the only way to quickly diagnose malaria disease remains RDTs. The choice of the SD Malaria Ag P.f/Pan® was based on the comparison of performance provided by the WHO reports [16, 17].

In FG, the implementation of malaria control strategies allowed to reduce the number of recorded cases by 82% during the last years [6, 19]. In 2011, among the 1209 reported cases, *P. falciparum* and *P. vivax* represented 31% and 68.5%, of cases, respectively [12]. In 2019, among the 212 recorded malaria cases, 6% were caused by *P. falciparum*, 42 cases were hospitalized, and only 2 cases developed a complicated clinical picture (one with *P. falciparum* and one with *P. vivax*) [20]*.* In the current study, the average share of confirmed *P. falciparum* malaria cases decreased significantly with 13.3% of positive tests (minus 17.7%), while confirmed *P. vivax* cases increased to 85.1% of positive tests (plus 16.6%)*.*

The current study analysed all performed RDTs and compared them to BFTs regardless of whether they were first-line diagnostic tests or monitoring tests during treatment. The global sensitivity of SD Malaria Ag P.f/Pan® test was 96% (88.2–1) for *P. falciparum* and 93% (90.6–94.2) for *P. vivax*. The sensitivity can be affected by a low rate of parasitaemia [17]. The global specificity was high, with a rate of 99.8% (99.5–1) for all species included. In our database, among 12,880 concomitant RDT and BFT, 10,873 (84.4%) represented first-line diagnosis tests, and 2007 RDTs were performed during the follow-up period. The latter affected results of the VVP, which was on average 97% (94.5–98.9), but significantly different for *P. falciparum* and *P. vivax* with 88% (80.4–1) and 98% (94.7–99.1), respectively.

Most of RDTs carried during the follow-up period were performed during the two first years of the study, which can explain the annual variation of the PPV. Furthermore, there is a significant variation of the global PPV week after week during the first 28 days of follow-up. It is also explained by the number of false positive cases related to the persistence of the protein-encoding for *Plasmodium* in the blood. In this study, we have not collected clinical information. Consequently, immunological factor or infectious agents explaining the calculated PPV were not investigated [21, 22]. Thus, a positive result of the RDT must be confirmed by a BFT. Otherwise, there is a risk of underdiagnosing malaria.

The NPV of SD Malaria Ag P.f/Pan® in the malaria diagnosis was, 99.7% (99.3–99.5) without a significant difference between *P. falciparum* and *P. vivax*. It has been reported that a low parasitaemia could affect the accuracy of the test with false-negative results [23], but this was not the case in this study. Consequently, the negativity of SD Malaria Ag P.f/Pan®, used as a first-line diagnosis test, allows to reasonably rule out malaria even though performing BFT remains compulsory.

In the current study, 2007 concomitant RDT/BFT were carried during the follow-up period of patients under treatment. The results have shown that the RDT remains positive up to 28 days, even though parasites were no longer detectable with BFT. Previous findings suggest that PfHRP2 RDTs remain positive after treatment for longer than the combined or pLDH RDTs [24]. This is explained by a slower degradation of PfHRP2 compared to pLDH after the parasite elimination [24, 25]. Indeed, PfHRP2 antigen, is progressively eliminated from the blood and can be responsible for false positives [26, 27]*.* Contrarily, pLDH is quickly eliminated from the blood within 1 week of treatment [28]*.* Thus, the initial parasite density influence false-positive results and PfHRP2 persistence [25].

Furthermore, RDTs have a higher probability of remaining positive in patients receiving artemisinin-based combination therapy (ACT) than in those receiving non-ACT drugs [25]. In practice, the persistence of positivity of RDT after treatment makes it irrelevant for monitoring. Thus, the SD Malaria Ag P.f/Pan® test should not be used to assess the efficacy of the treatment set up in these conditions.

During the last 2 years, the majority of malaria cases in FG were autochthonous (83% first-quarter 2019, 78% first-quarter 2020) while the remaining cases were from Brazil (range 9 to 14%), Suriname (range 2 to 5%), or Africa (range 2 to 7%) [7]. Illegal gold miners (*garimpeiros*) inside the rainforest and native populations on border areas between Suriname and Brazil are at risk of malaria resurgence [6, 29, 30]. The epidemic of malaria experienced a sharp increase in 2017 on the Amazonian border between FG and Brazil. Multifactorial causes were pointed out, such as migration from Brazil and Venezuela and related issues, local politics, logistics issues, amongst others [31]. Most cases occurred in forested areas except Saint Georges d' Oyapock on the Brazilian border [29, 32]. In FG, the malaria incidence is low, so all patients with positive RDT are considered as recently infected. However, in 2015, Orpal-1, a study carried out on a population of 421 gold miners, has shown that the wide majority of malaria cases were Brazilian citizens (93.8%). This study has shown that the malaria prevalence in asymptomatic carriers, researched by PCR, ranged from 22.3 to 84%. Species identified were *P. falciparum* and *P. vivax*, 47.9% and 37.2%, respectively with 10.6% co-infections. Thus, there is a risk of periodic reintroduction of the disease in FG [33]. It is estimated that illegal gold miners are around 10,000 people. Thus, Orpal-1 investigated only 4.2% of the garimpeiros and might not be representative.

It is worth noting that, in the Brazilian Amazon basin, the lack of PfHRP2 protein varies from region to region. It is the highest in Acre state (31.6%) and absent in Para state [34]. However, *P. falciparum* HRP3 deletion was recorded in 100% of cases [11]. In 2013, CDC reported PfHPR2 deletion in 14% of cases in Brazil, 14.1% in Suriname, 33.3% in Peru, 7.5% in Colombia and 4% in Bolivia [35]. Moreover, there is no accurate data about imported malaria cases in FG, the origin of immigration waves and if patients come from endemic countries with a significant rate of PfHPR2 deletion. In theory, this can represent a risk of false-negative tests that should be better estimated by further studies [36].

## Conclusion

SD Malaria Ag P.f/Pan® is a reliable rapid test used for the first-line diagnostic of malaria in remote healthcare centres in FG. However, it must no longer be used during the follow-up period of patients diagnosed with malaria. The test reading should be interpreted with caution while considering the recent medical history of patients and their arrival date in FG. BFT must always confirm RDT results. In FG, uncontrolled migratory flows might increase the risk of importing new variants of *Plasmodium* and impact the efficiency of the test.

## Data Availability

The datasets used and/or analysed during the current study are available from the corresponding author on reasonable request.

## Abbreviations

ACT: Artemisinin-based combination therapy
BFT: Blood Film Tests
CDC: Centers for Disease Control and Prevention
FG: French Guiana
FN: False negative
FP: False positive
IT: Information Technology
Nb: Number
NPV: Negative Predictive Value
PPV: Positive Predictive Value
RDT: Rapid diagnosis tests
Sp: Specificity
Ss: Sensitivity
TN: True negative
TP: True positive
WBC: White blood cells
WHO: World Health Organization
